# Clinical Aspects of Cutaneous Metastasis from Non-Cutaneous Primary Tumors

**DOI:** 10.3390/cancers17193126

**Published:** 2025-09-26

**Authors:** Michela Starace, Stephano Cedirian, Luca Rapparini

**Affiliations:** 1 Dermatology Unit, IRCCS Azienda Ospedaliero-Universitaria di Bologna, 40138 Bologna, Italy; 2 Department of Medical and Surgical Sciences, Alma Mater Studiorum, University of Bologna, 40138 Bologna, Italy

**Keywords:** cutaneous metastasis, solid cancer, hematological cancer, melanoma, lung cancer, breast cancer, dermoscopy, histopathology

## Abstract

Cutaneous metastases (CMs) represent an uncommon but clinically significant manifestation of advanced malignancies, originating from both solid and non-solid cancers. This review explores the clinical characteristics and prognostic implications of CMs. For solid cancers, CMs are most frequently associated with primary malignancies of the breast, lung, and gastrointestinal tract, presenting as nodules, plaques, or ulcerative lesions. In contrast, CMs from non-solid cancers, such as hematologic malignancies, often exhibit distinct patterns, including diffuse infiltrates or erythematous plaques, mimicking inflammatory dermatoses. Clinical features, as well as dermoscopy, may help, but diagnostic confirmation relies on histopathological evaluation and immunohistochemical studies, which are essential for determining the primary source of the malignancy. Clinically, CMs often signify a poor prognosis, necessitating prompt recognition and tailored management to improve patient outcomes. This comprehensive review aims to enhance clinical understanding and awareness of CMs to facilitate early diagnosis and optimized treatment strategies.

## Simple Summary

Cutaneous metastases represent the spread of internal malignancies to the skin. Although relatively uncommon, their recognition is clinically important, as they may constitute the first visible manifestation of an undiagnosed tumor or indicate advanced disease. The clinical appearance is highly variable, often mimicking benign or inflammatory skin conditions, which may delay correct diagnosis. Early identification is essential because these lesions are usually associated with an unfavorable prognosis and require prompt management. This review provides an overview of the clinical spectrum of cutaneous metastases, discusses the diagnostic tools available, and emphasizes the importance of awareness to facilitate timely recognition and optimize patient care.

## 1. Introduction

Cutaneous metastases (CMs), while relatively rare, carry significant prognostic implications and can occasionally serve as the first sign of an undetected internal malignancy [1,2]. Their clinical appearance often mimics more common dermatologic conditions—such as lipomas, cellulitis, epitheliomas, pyogenic granulomas, or inflamed and non-inflamed epidermoid cysts—leading to potential delays in diagnosis, particularly when the primary cancer remains occult [3,4,5,6,7,8]. These skin metastases most frequently arise from solid tumors, especially breast, lung, and gastrointestinal cancers, and typically present as firm, painless, erythematous nodules, plaques, or ulcerative lesions [9,10,11,12,13,14,15]. In contrast, hematologic malignancies that spread to the skin often manifest differently, with widespread infiltration or red plaques that resemble inflammatory skin diseases [16,17]. In women, breast cancer and melanoma are the most common sources of skin metastases, whereas in men, melanoma, and cancers of the head and neck, lung, and colon are more often implicated. Breast cancer, among all malignancies, is most prone to cutaneous dissemination [1,2,3,9,10,11,12]. Skin metastases may occur near the site of the primary tumor and generally exhibit histologic characteristics that resemble, though not always exactly match, the primary neoplasm [3,18,19,20,21,22]. Due to their association with poor outcomes, recognizing the broad clinical and histopathological spectrum of CMs is essential. Importantly, this review does not focus on melanoma satellite or in-transit metastases (ITMs), for which there already exists a robust literature and which are relatively common in melanoma. Although ITMs are mentioned for context, the scope of this article is to highlight the far less common but clinically relevant phenomenon of CMs arising from internal malignancies.

## 2. Epidemiology

CMs from solid tumors, though relatively uncommon, represent a significant clinical finding that often signals advanced systemic disease. Their overall incidence ranges from 0.7% to 9%, and this number appears to be rising in parallel with the improved survival of patients with solid malignancies [2,9,10]. CMs tend to occur more frequently in individuals aged between 50 and 70 years, with a slight male predominance in most series, although female predominance has been noted in cohorts with a high representation of breast cancer [2,9,10]. Pediatric cases are rare and often linked to rhabdomyosarcoma and neuroblastoma (Table 1) [3,11,19].

The likelihood of skin involvement varies by cancer type—metastatic melanoma has the highest rate, with cutaneous spread occurring in about 2–20% of cases [1,2,3,9,10,11,12,18,19,20,21]. Sex-specific prevalence patterns also influence the distribution: in women, CMs most commonly originate from breast cancer (accounting for 70%), followed by melanoma and tumors of the ovary, head and neck, and lung. In men, melanoma is the leading cause (32%), followed by carcinomas of the head and neck (16%), lung (12%), and colon (11%) [1,2,3,9,10,18]. The overall prevalence of a malignancy also affects how frequently it contributes to CMs; for example, although only 2.5% of men with metastatic lung cancer develop skin lesions, lung cancer still represents 12% of male CMs due to its high incidence [1,2,3,9,10,11,12].

Temporal patterns of appearance also vary [23]. Most CMs are diagnosed metachronously—that is, after the identification of the primary tumor. Sixty percent of metastases occurred in a metachronous pattern, while 32% were synchronous and only 8% were diagnosed early, sometimes even preceding the detection of the primary tumor [2]. The latter scenario, although less common, has been documented in several tumor types. For instance, the 17.6% of prostate cancer CMs preceded the diagnosis of the primary tumor by an average of nearly 11 months [24]. Rectal cancer has also been reported to present initially with skin metastases in rare cases, especially when involving extensive perineal soft tissue [25,26]. Cases of metastatic thyroid cancer without an identifiable primary tumor in the thyroid are also described [27].

Regarding the epidemiology of CMs due to hematological malignancies, they account for approximately 20–50% of reported cutaneous lymphoma cases, though this wide range is likely influenced by the retrospective nature and referral bias of existing studies [17,28,29,30,31,32]. Skin involvement in Hodgkin lymphoma (HL) is notably rarer, ranging from 0.5% to 7% of cases and it typically affects men in their 5th decade [33,34]. A major study from South Korea analyzing 106 cases of secondary cutaneous lymphomas found that B-cell lineage lymphomas were more common in older individuals compared to T/NK-cell variants, although there was no sex difference or distinct pattern in primary tumor location [32]. While lymph nodes were the most frequently involved sites at diagnosis, T/NK lymphomas were more likely to show extra-nodal spread and bone marrow involvement.

Leukemia cutis is an uncommon manifestation of systemic leukemia, and precise data regarding its overall incidence and demographic distribution remain lacking. Nonetheless, it has been observed that children with congenital leukemia exhibit a relatively high propensity to develop leukemia cutis, with reported rates ranging from 25% to 30% of cases [35]. Although the highest incidence of leukemia cutis is associated with adult T-cell leukemia/lymphoma (ATLL), this hematologic malignancy itself is exceedingly rare. Consequently, the most frequently encountered subtypes in clinical practice are acute myeloid leukemia (AML) and chronic lymphocytic leukemia (CLL), accounting for approximately 13% and 8% of leukemia cutis cases, respectively [35]. In the majority of patients (55% to 77%), cutaneous manifestations occur after the diagnosis of systemic leukemia. However, skin lesions may also present concurrently with systemic disease (23% to 44%) or, more rarely, may precede detectable hematologic abnormalities in the peripheral blood or bone marrow (2% to 3%) [35]. This latter presentation, referred to as "aleukemic" leukemia cutis, typically progresses to AML [36].

## 3. Pathogenesis

Metastasis refers to the formation of a tumor in a location distant from the original (primary) site of malignancy [10]. The metastatic spread of cancer involves a complex multistep process where tumor cells detach from the primary site, enter the circulation, survive transit, exit into new tissue, and eventually proliferate [37,38,39]. Despite the frequent shedding of malignant cells into the bloodstream, only a minute fraction—estimated at ≤0.01%—successfully form secondary tumors [3]. Two principal models explain how malignancies gain metastatic capacity. The linear progression model suggests that genetic mutations accumulate over time, enabling certain tumor clones to metastasize, resulting in metastatic lesions that closely resemble the primary tumor [38,39]. Conversely, the parallel progression model posits that rare, stem-like tumor cells with high metastatic potential may disseminate early and evolve independently [39].

Metastases often target specific organs, influenced by both mechanical factors and molecular cues such as adhesion molecules (e.g., integrins), which help tumor cells anchor and invade tissues [3,18,19,20,21]. Equally important is the contribution of tumor-associated lymphangiogenesis, which facilitates access of malignant cells to lymphatic vessels and plays a pivotal role in progression to sentinel lymph nodes, often anticipating systemic dissemination and correlating with poor prognosis [40]. In fact, lymphangiogenesis may also occur at distant metastatic sites, where it promotes further spread of tumor cells to other organs [40]. For instance, lesions on the extremities often indicate arterial embolization, while widespread skin involvement suggests systemic hematogenous dissemination. In contrast, metastases near the primary tumor usually point to lymphatic or venous spread, with retrograde lymphatic flow sometimes explaining perineal or lower limb involvement from pelvic tumors [3,18,19,20,21]. The head, neck, and upper trunk are commonly affected, likely due to their rich vascular supply. Specific sites such as the umbilicus (e.g., Sister Mary Joseph nodule, typically from gastrointestinal cancers), surgical scars, or irradiated skin areas may also harbor metastases [3,18,19,20,21,41,42,43]. Scar involvement usually reflects local lymphatic or venous spread, though rare cases of tumor localization to distant or recent surgical sites—including skin graft donor areas—have also been observed [21,44]. Clinical and experimental evidence further suggests that iatrogenic damage to lymphatic vessels—such as that occurring during extensive lymph node dissection or reckless flap surgery—can compromise local immune surveillance, thereby facilitating tumor growth and metastatic progression [45]. Emerging research suggests that tumor-derived exosomal non-coding RNAs may play a role in directing metastatic tropism, although the full mechanisms remain under investigation [21].

## 4. Clinical Features

### 4.1. Solid Malignancies

CMs present with diverse clinical features that may assist diagnosis, especially in patients with known cancer (Table 2). Clinically, the most common presentation of CMs is a dermal or subcutaneous nodule. In a Spanish tertiary hospital series, 81% of patients with CMs presented with nodular lesions [2]. Similarly, nodules were the predominant morphology in cases of CMs from prostate cancer, comprising 79% of presentations [24], and were also reported in 63.6% of gastric cancer CMs included in a systematic review of the literature [12]. Plaques and papules are also seen, though less frequently. Breast cancer, in particular, was noted to manifest more often as plaques rather than nodules, in contrast to CMs from other primary sites [1,2,3]. Papular and plaque-like presentations were also identified in gastric cancer, accounting for 6.1% and 21.2% of cases, respectively [12].

The anatomical distribution of CMs is influenced by the primary tumor site and by patterns of lymphatic or hematogenous dissemination [1,2,3,9,10,11,12,13,18,19,20,21,24,25,26]. Spread can occur via hematogenous, lymphatic, or direct extension, with proximity to the primary site often offering a diagnostic clue—for example, in-transit melanoma or direct invasion from breast or head and neck carcinoma [21]. The scalp and anterior thorax are among the most commonly involved regions [1,2,3,9,10,11,12,18,19,20,21,24,25,26]. Scalp, trunk, and axillary regions are frequently affected, particularly in lung and breast cancer [2]. Likewise, breast cancer tends to metastasize to the anterior thorax, while gastrointestinal cancers, including colorectal and gastric neoplasms, more often involve the abdomen and umbilical region [10]. The umbilical site is very rare and classically associated with gastrointestinal and gynecologic primaries and may manifest as the so-called Sister Mary Joseph nodule [10,12,41]. In rare cases, CMs can occur in less typical sites such as the lower limb, as illustrated by a case of urothelial bladder carcinoma with a metastasis to the right leg [46]. Cases of subungual metastasis are also described [47,48,49]. Direct skin invasion may also cause atypical manifestations such as dermal sclerosis, telangiectasia, peau d’orange, or erysipeloid spread, usually near the primary tumor, and often requiring a high level of clinical suspicion for correct identification [50,51,52,53,54,55,56,57,58,59,60]. Breast cancer has the broadest cutaneous spectrum, from nodules and erysipelas-like plaques to woody, indurated en cuirasse presentations. Unusual patterns—like vascular papules from intravascular spread or zosteriform distributions—further complicate diagnosis [50,51,52,53,54,55,56,57,58,59,60,61,62]. Some of these peculiar patterns of presentation are as follows:

#### 4.1.1. Carcinoma en Cuirasse

Carcinoma en cuirasse typically evolves from an early inflammatory phase, sometimes with nodularity, into a sclerodermoid plaque resembling morphea. In patients with prior breast cancer treated with radiotherapy, distinguishing this condition from post irradiation morphea can be challenging—particularly during its underrecognized inflammatory onset, which may mimic recurrence and raise clinical concern [23,50,51,52,53,54,61]. While most often associated with breast cancer, carcinoma en cuirasse has also been reported in malignancies of the lung, gastrointestinal tract, kidney, and others [50,51,52,53,54,63].

#### 4.1.2. Carcinoma Erysipeloides

**Figure 1:**
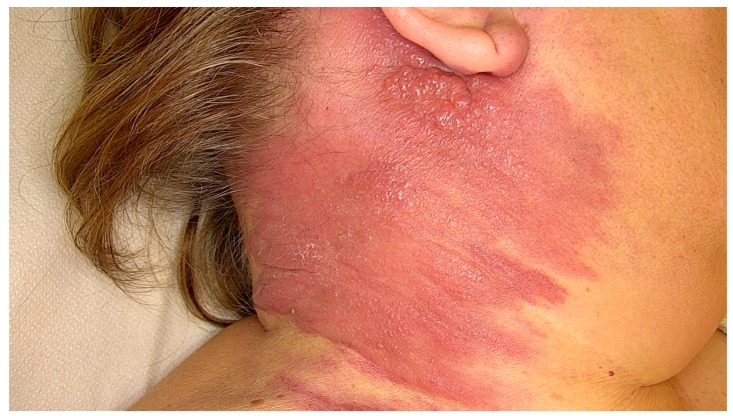
Carcinoma erysipeloides from lung cancer. Warm, indurated erythematous plaques on the neck mimic erysipelas, due to dermal lymphatic tumor emboli.

Carcinoma erysipeloides mimics erysipelas, presenting as a warm, tender, edematous plaque but notably without fever or systemic signs (Figure 1) [23,55,56,57,63]. This pattern is most frequently seen in breast cancer and accounts for nearly one-third of its CMs [55,56,57,61]. However, similar clinical features have been observed in other malignancies, including melanoma, mesothelioma, and various carcinomas such as those of the lung, prostate, bladder, colon, and pancreas [55,56,57]. The condition typically results from dermal lymphatic obstruction by tumor emboli, rather than direct lymphatic invasion, with histology revealing carcinoma cell clusters occluding dermal lymphatics at multiple levels [55,56,57].

#### 4.1.3. Teleangiectatic Metastatic Carcinoma

Telangiectatic metastatic carcinoma, most commonly linked to breast cancer, can be diagnostically challenging due to the subtlety of the vascular changes and the often-sparse presence of tumor cells, occasionally mimicking angiosarcoma [23,58,59].

#### 4.1.4. Sister Mary Joseph Nodule

Sister Mary Joseph’s nodule refers to a palpable nodule at the umbilicus, typically indicating metastatic spread from an intra-abdominal or pelvic malignancy. It is most often linked to advanced-stage disease with peritoneal involvement and generally signifies a poor prognosis [60].

#### 4.1.5. Less Typical Presentation

Other less typical presentations include intertrigo-like lesions in the inframammary fold, and nodular eruptions due to lymphatic obstruction from pelvic tumors [3,18,19,20,21]. Local or in-transit metastases, particularly from melanoma, are also notable, especially following interventions such as biopsies, drainage of malignant effusions, or surgical procedures. In these cases, metastasis can result from direct tumor seeding—so-called “tumor spillage”—although current rates of surgical wound or port site metastases are low, around 0.8% [9,10,18,19,20,21].

### 4.2. Hematological Malignancies

#### 4.2.1. General Aspects of Hematological Malignancies

Beyond metastases originating from solid tumors, cutaneous manifestations can also result from leukemias and systemic lymphomas [16,17,64]. These lesions frequently appear as papules or nodules with hues ranging from pink-violet to red-brown, making them clinically difficult to distinguish from skin metastases caused by solid cancers (Table 3) [17,29,30,31,32,34,65,66]. Although the term "metastasis" is generally not applied to skin infiltration by leukemia or lymphoma, these lesions will nonetheless be reported due to their significant prognostic implications, as they represent true cutaneous dissemination of internal hematologic malignancies [3,21].

#### 4.2.2. Lymphomas

Skin involvement in lymphomas can appear in many forms, such as papules, plaques, tumors, or even erythroderma, as reported in several studies [16,17,28,29,30,31,32,34,65,67,68,69,70]. Notably, Lee and colleagues found that lymphomas of the T/NK-cell type tend to manifest with maculopapular lesions, while B-cell skin-confined lymphomas are more commonly associated with plaques or nodular formations [17,32,65,67,68,69]. Certain subtypes, including angioimmunoblastic lymphoma (AIL) and peripheral T-cell lymphoma not otherwise specified (PTNL), may clinically resemble inflammatory skin conditions, morbilliform rashes or erythroderma [34,65,67,68,69]. HL frequently presents as reddish-brown papules, patches, or nodules, which may occasionally ulcerate [16,17].

In the spectrum of T/NK-cell derived lymphomas, those most frequently linked to cutaneous signs include PTNL, NK/T-cell lymphoma, anaplastic large-cell lymphoma (ALCL), AIL, and ATLL. Some of these lymphomas can extend into subcutaneous tissues [17,32]. It is therefore essential to distinguish them from primary subcutaneous T-cell lymphomas—a category defined by its localization to subcutaneous tissue, specific immunophenotype (T-cytotoxic alpha/beta), and typically favorable clinical outcome [17,70]. Anatomically, T/NK-cell CMs tend to involve the extremities, B-cell cutaneous metastasis the trunk, and HL the head, neck, axillary, and inguinal regions, often reflecting regional lymphatic drainage [17,32].

#### 4.2.3. Leukemia Cutis

Leukemia comprises several subtypes, each with distinct age-related patterns [71]. Acute lymphoblastic leukemia (ALL) primarily affects children, whereas AML and chronic myeloid leukemia (CML) are more prevalent in adults [3,37,71]. CLL and hairy cell leukemia are most frequently diagnosed in older individuals [3,37,71]. Leukemia cutis-specific infiltration of leukemic cells into the skin—may signify disease progression. In CML, the onset of leukemia cutis can signal transformation into the blast phase. Similarly, in patients with myelodysplastic syndromes (MDS), the appearance of leukemia cutis often indicates evolution toward AML. Some individuals with MDS may also present with indolent cutaneous lesions composed of benign-looking dendritic or myeloid cells. Among all leukemia types, AML—particularly with NPM1 mutations—is most commonly associated with leukemia cutis, while CLL less frequently shows this manifestation [21,71,72,73,74,75,76,77,78,79]. Leukemia can also cause nonspecific reactive skin eruptions or more distinct leukemic infiltrates [72,73,74,75,76,77,78].

Specific cutaneous manifestations generally appear as multiple firm papules, plaques, or nodules, localized or widespread, with colors ranging from pink to red-brown or purple [36,37,72,73,74,75,76,77,78,79]. Macules, ulcerative lesions, blisters, and palpable purpura are more exceptional [36]. These lesions may bleed, a complication often linked to thrombocytopenia. In rare cases, ulcerated or bullous lesions develop [72,73,74,75,76,77,78,79]. The most affected sites include the head, neck, and trunk, although lesions can occur anywhere. In some cases, leukemic infiltrates emerge in areas of previous trauma or scars [72,73,74,75,76,77,78,79]. On rare occasions, patients with myeloid leukemias present with subcutaneous or dermal nodules known as myeloid sarcomas or chloromas. These can precede the onset of systemic disease by several months [3]. In a study examining skin biopsies from leukemic patients, leukemia-specific eruptions accounted for around 30% of cases [79]. Cutaneous lesions typically appear at the time of leukemia diagnosis or during relapse, but in rare instances, they may precede hematologic findings—a phenomenon referred to as “aleukemic” leukemia cutis [37,75]. Occasionally, these lesions emerge months or even years before bone marrow involvement becomes evident [37,75]. In CLL, asymptomatic dermal infiltration by leukemic cells may be incidentally found in biopsies of unrelated skin conditions, such as squamous cell carcinoma or herpesvirus infections (e.g., HSV or VZV). This reflects the leukemic cells’ ability to migrate toward inflammatory signals—a process known as inflammatory oncotaxis [73,80].

## 5. Clinical Features of Special Sites

### 5.1. Scalp Metastases

The scalp is a distinct anatomical site, involved in ~2% of skin cancers, with CMs accounting for 4–7%, likely due to its rich vascular supply [81]. Scalp metastases (SMs) most often originate from GI tract, lung, breast, prostate, and kidney [81,82]. In a review of 123 patients, a slight female predominance (53.7%) is described, likely due to breast cancer rates; GI tract was the leading primary source (24.4%), followed by breast (17.9%), kidney (8.1%), lung/thyroid (7.3%), uterus/CNS (6.5%), liver (3.3%), and others (18.7%) [83]. A multicenter study on 583 patients confirmed the head and neck as common sites for CMs, especially from melanoma and breast cancer [84]. Literature remains limited, mostly to case reports and small series [42,44,81,83,84,85,86]. The term "neoplastic alopecia" is sometimes used for SMs but can be misleading since SMs show various lesion types and may not involve alopecia. True neoplastic alopecia typically presents as diffuse hair loss without patches, requiring distinction through clinical and histologic evaluation [81,87].

**Figure 2:**
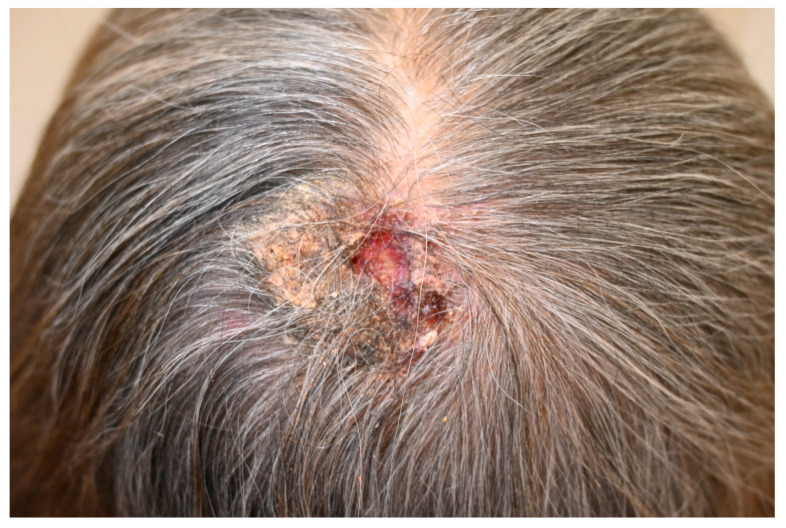
Scalp metastasis from breast carcinoma. Ulcerated nodular lesion with crusting and scarring alopecia on the vertex, consistent with neoplastic scalp involvement.

SMs show diverse clinical features influenced by lesion type, size, color, location, and associated skin findings. Morphologically, SMs may appear as papules, plaques, nodules, ulcers, or inflammatory eruptions, often asymptomatic, pruritic, or painful (Figure 2). They are usually solitary but can be multiple. Eleven subtypes of SMs have been identified based on appearance, with red/violaceous nodules (average 2.2 cm) and flesh-colored nodules (average 3.4 cm) being most frequent; red lesions predominate in the parietal scalp, while uncommon variants are observed in frontal/parietal/occipital areas [83]. SMs can be associated with other skin manifestations related to the primary tumor (e.g., telangiectasias, zosteriform eruptions) and with secondary neoplastic alopecia (SNA), a scarring alopecia likely caused by tumor-induced desmoplastic reaction and cytokine-mediated follicular atrophy (e.g., TGF-β, IL-4, IL-6, bFGF) [81].

### 5.2. Oral Mucosal Metastases

Mucosal metastases to the oral cavity are uncommon but clinically significant, often representing the first manifestation of an undiscovered malignancy in up to 30% of cases [88,89,90,91]. The attached gingiva is the most frequently involved site among oral soft tissues, accounting for over 50% of mucosal metastases, followed by the tongue and buccal mucosa [89,90]. Clinically, these lesions often present as painless, exophytic nodules or masses that may be ulcerated or erythematous and are frequently misdiagnosed as reactive or hyperplastic conditions such as pyogenic granuloma or peripheral giant cell granuloma [89,90]. In some cases, especially when adjacent bone is affected, symptoms such as tooth mobility, bleeding, dysphagia, and masticatory discomfort may be present [90]. Histologically, the majority of mucosal metastases are carcinomas, including adenocarcinomas and squamous cell carcinomas, and immunohistochemistry is often required to determine the primary origin, particularly when no prior diagnosis of malignancy exists [91]. Lung cancer is the most common primary tumor in males with mucosal metastases, while breast cancer is most frequent in females [89,90]. Other common primary sites include the kidney, colorectum, and liver, with site-specific predilections noted; for example, renal and cutaneous tumors more frequently metastasize to soft tissues [90]. When gingival metastases are accompanied by underlying alveolar bone destruction, radiographs may reveal irregular radiolucent lesions with ill-defined borders, although these findings are non-specific and may mimic odontogenic infections or inflammatory bone disease [90,91]. Importantly, mucosal metastases are associated with poor prognosis: median survival following diagnosis of oral soft tissue metastasis is approximately 5 to 9.8 months [88,89,90,91].

## 6. Dermoscopy

**Figure 3:**
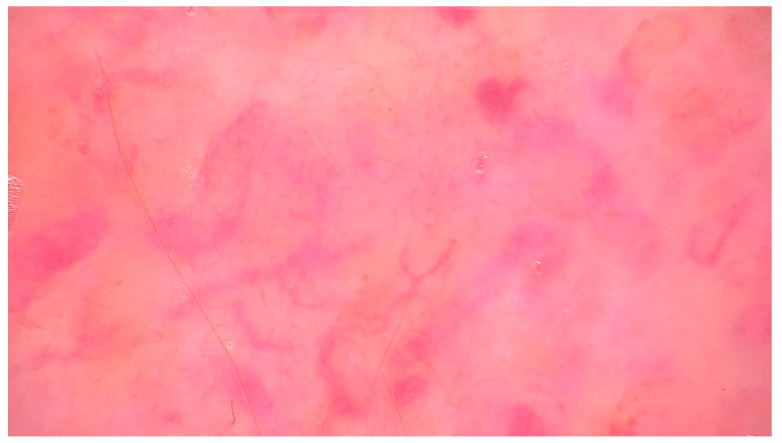
Dermoscopy of cutaneous metastasis showing polymorphous vessels. Polymorphous vessels (linear irregular, serpentine, and hairpin) on a pink-purple background in a right arm lesion of a 69-year-old man with metastatic prostate carcinoma.

Although CMs often lack pathognomonic clinical features, dermoscopy can provide important visual clues that raise suspicion and support the decision to biopsy. One of the most relevant dermoscopic findings is the presence of atypical vascular structures, frequently arranged in a disorganized and polymorphous pattern (Figure 3) [82,84,92,93]. These include combinations of linear, irregular, dotted, serpentine, and hairpin vessels, often lacking symmetry and uniform distribution. Such vascular atypia reflects the neoangiogenesis and structural disarray typical of metastatic dermal infiltration [84,92].

Another recurring observation under dermoscopy is the presence of structureless pink to whitish areas, sometimes interspersed with shiny white lines visible under polarized light. These areas correspond histologically to dense tumor cell aggregates and dermal fibrosis, common features of metastatic skin involvement. Notably, pigmented structures such as networks, globules, or streaks are typically absent, which helps differentiate metastases from melanocytic lesions like melanoma [84,92,93].

Ulceration and hemorrhagic crusts may also be observed, particularly in lesions that are rapidly enlarging or mechanically traumatized, further contributing to a non-specific but suspicious dermoscopic profile [84,92]. In some cases, subtle correlations between dermoscopic appearance and the type of primary tumor have been proposed. For instance, breast carcinoma metastases may present with serpentine vessels on a pink-white background, while metastases from gastrointestinal or renal primaries might exhibit prominent vascularity or coiled vessels. However, such associations are not consistent enough to allow tumor-type prediction based on dermoscopy alone [84].

Overall, while dermoscopy cannot establish a definitive diagnosis of cutaneous metastasis, it plays a valuable role in differentiating these lesions from benign entities such as cysts, dermatofibromas, or vascular tumors [81,84,92,93]. In the context of a new, firm, or atypically vascularized skin lesion—particularly in a patient with known cancer history—dermoscopic suspicion should prompt histopathologic evaluation.

## 7. Conclusions

CMs, although relatively uncommon, represent a critical clinical indicator of advanced systemic malignancies. Their diverse presentations—ranging from nodules and plaques to erysipeloid and en cuirasse patterns—may mimic inflammatory dermatoses, posing a diagnostic challenge. Solid tumors such as breast, lung, and gastrointestinal cancers remain the most frequent sources, but hematologic malignancies, including leukemias and lymphomas, contribute a significant share and exhibit unique clinical patterns. Special anatomical sites such as the scalp and oral mucosa further underscore the heterogeneity of CMs manifestations. Dermoscopy may aid in raising suspicion, but histopathological and immunohistochemical confirmation are essential for diagnosis and management. Recognizing the broad clinical spectrum of CMs is crucial for timely diagnosis, prognostic assessment, and the initiation of appropriate oncologic treatment. Enhanced awareness among clinicians—particularly dermatologists and oncologists—can lead to earlier detection and potentially improved outcomes for patients with underlying malignancies.

## Figures and Tables

**Table 1:** Most common primary tumors associated with CMs.

Sex	Most Frequent Primary Tumors	Relative Frequency (%)	Most Common Cutaneous Sites
Adult female	Breast, melanoma, ovary, head & neck, lung	Breast up to 70%	Anterior chest, scalp
Adult male	Melanoma, head & neck, lung, colon, prostate	Melanoma ~32%; lung ~12%; colon ~11%	Scalp, trunk, abdomen
Children	Rhabdomyosarcoma, neuroblastoma	Rare	Variable, often multiple

CMs: cutaneous metastases.

**Table 2:** Main clinical features of CMs from solid tumors.

Clinical Presentation	Typical Primary Tumor	Distinctive Notes
Dermal/subcutaneous nodules	Lung, colon, kidney, prostate	Most common form (60–80%)
Indurated plaques	Breast, gastric	Breast (frequent en cuirasse)
Carcinoma en cuirasse	Breast, lung, GI	Sclerodermiform aspect, chest involvement
Carcinoma erysipeloides	Breast (~30% of CMs), lung, prostate	Erythematous warm plaques, no fever
Telangiectatic metastatic carcinoma	Breast	May mimic angiosarcoma
Sister Mary Joseph nodule	GI and gynecological tumors	Umbilical localization, poor prognosis
Rare patterns (zosteriform, subungual, intertrigo-like)	Various	May mimic inflammatory dermatoses

CMs: cutaneous metastases; GI: gastrointestinal.

**Table 3:** Clinical features of CMs from hematologic malignancies.

Subtype	Prevalent Morphology	Typical Localization	Distinctive Notes
T/NK-cell lymphomas	Maculopapules, morbilliform rash, erythroderma	Extremities	May mimic inflammatory dermatoses
B-cell lymphomas	Nodules/plaques	Trunk	Single or multiple lesions
Hodgkin lymphoma	Reddish-brown papules/nodules	Head–neck, axillary, inguinal	Rare (0.5–7%)
Leukemia cutis (AML, CLL)	Erythematous-violaceous papules/nodules, sometimes ulcerated	Head–neck, trunk	May precede hematologic diagnosis (2–3%)
Aleukemic leukemia cutis	Infiltrated nodules/plaques	Variable	Precedes peripheral blood involvement

ALL: acute lymphoblastic leukemia; CLL: chronic lymphocytic leukemia; CMs: cutaneous metastases.
